# PIGD: a database for intronless genes in the Poaceae

**DOI:** 10.1186/1471-2164-15-832

**Published:** 2014-10-01

**Authors:** Hanwei Yan, Cuiping Jiang, Xiaoyu Li, Lei Sheng, Qing Dong, Xiaojian Peng, Qian Li, Yang Zhao, Haiyang Jiang, Beijiu Cheng

**Affiliations:** Key Laboratory of Crop Biology of Anhui Province, Anhui Agricultural University, Hefei, 230036 China

**Keywords:** Poaceae, Intronless Genes, Database

## Abstract

**Background:**

Intronless genes are a feature of prokaryotes; however, they are widespread and unequally distributed among eukaryotes and represent an important resource to study the evolution of gene architecture. Although many databases on exons and introns exist, there is currently no cohesive database that collects intronless genes in plants into a single database.

**Description:**

In this study, we present the Poaceae Intronless Genes Database (PIGD), a user-friendly web interface to explore information on intronless genes from different plants. Five Poaceae species, *Sorghum bicolor*, *Zea mays*, *Setaria italica*, *Panicum virgatum* and *Brachypodium distachyon*, are included in the current release of PIGD. Gene annotations and sequence data were collected and integrated from different databases. The primary focus of this study was to provide gene descriptions and gene product records. In addition, functional annotations, subcellular localization prediction and taxonomic distribution are reported. PIGD allows users to readily browse, search and download data. BLAST and comparative analyses are also provided through this online database, which is available at http://pigd.ahau.edu.cn/.

**Conclusion:**

PIGD provides a solid platform for the collection, integration and analysis of intronless genes in the Poaceae. As such, this database will be useful for subsequent bio-computational analysis in comparative genomics and evolutionary studies.

## Background

In eukaryotes, most genes are composed of exons and introns. Exons are the coding regions of genes that are transcribed and collectively form a contiguous coding sequence. Introns, commonly found in eukaryotes between exon sequences in the DNA, are removed from RNA molecules through splicing during post-transcriptional RNA processing. Although the exon–intron gene structure, the split structure of genes, is typical, intronless or single-exon genes account for a significant proportion in most eukaryotes. Intronless genes are a characteristic feature of prokaryotes and an important resource for understanding the rules of gene structure and organization, protein functionality and evolutionary differences between eukaryotes and prokaryotes. With the availability of complete genome sequences, several studies have attempted to identify intronless genes in many eukaryotic genomes and subsequently determine their functional and evolutionary history. To date, 2,017 (~8%) intronless genes have been identified in mice [1]; 930–6,229 (7.9%–16.7%) in deuterostomes [2]; 542–1,014 (2.8%–4.5%) in teleost fish [3]; 11,109 (19.9%) in rice; and 5,846 (21.7%) in *Arabidopsis thaliana* [4]. These intronless genes encode proteins from a variety of families, including G-protein–coupled receptors (GPCRs) [5, 6], olfactory receptors [7, 8] and small auxin-up RNAs (SAURs) [9], which are essential for various biological functions. Data show that proteins encoded by intronless genes participate in growth regulation, cell proliferation, development, sperm formation and immune responses [10–12]. The origin of intronless genes is not clear and represents a fascinating area of research, partly because of their prokaryote-like structure. It has been suggested that intronless genes form when introns are lost; this would generate a subset of intron-free genes [13]. Another possibility is that intronless genes evolved as a result of reverse transcription [14]. In the process of retroposition, mRNAs are reverse-transcribed into cDNA and inserted into new genomic positions that lack introns [15].

To date, three intronless gene databases have been created, including SEGE for *Homo sapiens*, *Drosophila melanogaster*, *Saccharomyces cerevisiae*, *Caenorhabditis elegans* and *Arabidopsis thaliana* genes [16]; Genome SEGE for eukaryotic genome sequences from NCBI [17]; and IGD for human intronless genes [18]. Currently, IGD is the only intronless gene database available on the Internet. Until now, no intronless gene database has been developed for sequenced Poaceae genomes.

With this in mind, we present the Poaceae Intronless Genes Database (PIGD; http://pigd.ahau.edu.cn/). This database, with a user-friendly web interface, provides access to a collection of intronless genes from five genome sequenced Poaceae, including *Sorghum bicolor*, *Zea mays*, *Setaria italica*, *Panicum virgatum* and *Brachypodium distachyon*. PIGD will allow researchers to easily identify intronless genes, integrate functional and evolutionary annotations, and search and download detailed information. In contrast to databases dedicated to individual organisms, the PIGD provides comparative analyses of genomic data from five species, and in conjunction with the BLAST program, it will allow users to explore and analyze data generated from the PIGD efficiently. We anticipate that PIGD will become a useful resource for the research community, particularly for studies of molecular function and the evolution of intronless genes.

## Construction and content

### Data sources

Currently, PIGD includes the following five organisms - *Sorghum bicolor*, *Zea mays*, *Setaria italica*, *Panicum virgatum* and *Brachypodium distachyon*. All genome data were downloaded from the Phytozome database (release version 9.1, http://www.phytozome.net/IEsorry.php?refer=/) [19].

### Identification of Poaceae intronless genes

A stringent protocol was used to reliably identify Poaceae intronless genes. First, using the Java program, we extracted genes that contain a “CDS” line in the GFF3 format files. Redundant sequences representing the same gene loci were then excluded. Finally, we retained sequences assembled into chromosomes for *Sorghum bicolor*, *Zea mays* and *Brachypodium distachyon*. Organelle sequences were discarded to avoid ambiguity. Scaffolds that could not be physically anchored in *Setaria italica* and *Panicum virgatum* were present in the data set. Thus, lists of non-redundant intronless genes from the five Poaceae organisms were generated.

### Data assembly

Each entry into PIGD integrates a comprehensive list of annotations on: (i) physical location, strand, protein length from Phytozome; (ii) gene family from Pfam [20] (http://pfam.sanger.ac.uk/); (iii) isoelectric point (PI) and molecular weight (Mw) from Expasy [21] (http://www.expasy.org/); (iv) GI number and definition from the NCBI protein project (http://www.ncbi.nlm.nih.gov/protein/); (v) a prediction of function based on cellular role and Gene Ontology (GO) from Protfun [22] (http://www.cbs.dtu.dk/services/ProtFun/); (vi) prediction of subcellular localization from WoLF PSORT [23] (http://www.genscript.com/psort/wolf_psort.html) and (vii) taxonomic distribution among different groups (archaea, bacteria, metazoans, fungi, plants, viruses and other eukaryotes) using Blink [24] (http://www.ncbi.nlm.nih.gov/sutils/blink.cgi?mode=query).

### Comparisons between Poaceae species

The records contained within the “Comparison” menu are grouped into eight general classes of related information: (i) the number and percentage of intronless genes; (ii) the distribution of PI; (iii) the distribution of Mw; (iv) the distribution of protein length; (v) the distribution of cellular roles; (vi) the distribution of GO categories; (vii) the number and percentage of species-specific intronless genes; and (viii) the gene size of the species-specific intronless genes.

### The PIGD web interface

A web-based platform, PIGD combined the MySQL (version 5.5.8) database management system with a dynamic web interface based on PHP (version 5.3.3) and Javascript (version 1.2).

## Utility

The PIGD currently contains 54,336 intronless genes from five sequenced Poaceae species (Table 1). Protein and transcript sequences were downloaded from Phytozome. The lack of comprehensive and consistent intronless gene databases means that it was essential to integrate information from all possible sources. Hence, we chose several representative data sources and analytic tools, including Pfam, Expasy, NCBI, Protfun and WoLF PSORT, to help aid the overall understanding of the function and evolution of intronless genes. Intronless gene sequences and annotation data were stored in a MySQL relational database for efficient retrieval of data from indexed files.

**Figure 1:**
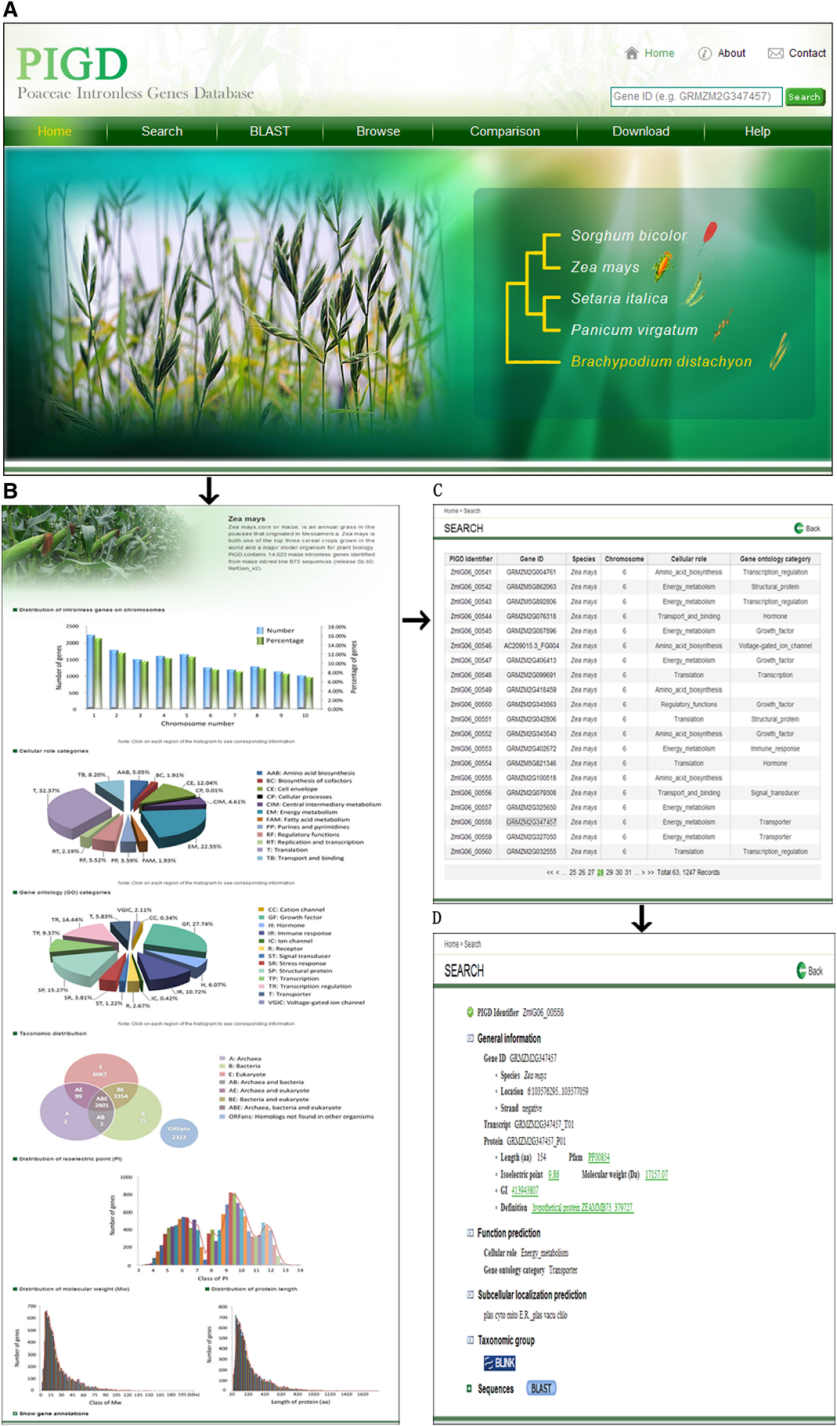
**An overview of the website and gene annotation page in the PIGD. (A)** The Home page. **(B)** The summary data for intronless genes found in *Zea mays*. **(C)** An example list of intronless genes that can be produced for a specific organism. **(D)** An example of the gene annotation page.

The web interface of PIGD was designed to include the following components: Home, Search, BLAST, Browse (for the database), Comparison, Download and Help. An illustration of the PIGD system is shown in Figure 1.

**Table 1:** **The number of intronless genes reported for each species**

Species	The number of intronless genes
*Sorghum bicolor*	6,197
*Zea mays*	14,623
*Setaria italica*	10,015
*Panicum virgatum*	17,227
*Brachypodium distachyon*	6,274

### Home

The user is introduced to the objective of the PIGD, the major scientific contributions, and the species that are currently included in the database. Details about the organization that developed the PIGD and contact information are also provided at the top of the home page.

### Search

There are two different ways to search the database: quick and advanced searches. Users can either type a truncated version or the entire ID for a gene, transcript and protein and PIGD identifier into the search field found on the top right of each page. Alternatively, an advanced search can be conducted, where the user is able to assign the species, chromosome, cellular role, GO category and Gene ID of interest, by clicking on the “Search” button on the home page. The results pages matching the query conditions are then shown. Finally, users are able to easily navigate from their search results to pages that contain the detailed annotations.

### Blast

BLAST search (release version 2.2.9) can be conducted from http://pigd.ahau.edu.cn/BLAST.php to search PIGD using DNA or protein sequence(s) as the query(s). Users can apply five BLAST search programs (blastn, blastp, blastx, tblastn and tblastx) with an E-value ≤ 1e-5 to find the putative homologous sequences of these intronless genes in different species.

### Browse

From the home page, users can click on “Browse” to select their desired species from the drop-down menu or by clicking directly on the image of the species seen in the middle of the home page. A page providing general information, including an overview of the species, the number of intronless genes, and chromosomal distribution, is then shown. In addition, functional categorization (cellular role and GO category); classification of intronless genes into the three super kingdoms of life (archaea, bacteria and eukaryotes); the distribution of PI, Mw and protein length; and the sources of all primary gene annotations are provided. Users are then able to click on part of the image from the “Browse” page to open a new window with gene entries that meet the specific conditions. An individual gene annotation displays basic information related to the gene description, including the physical location and chromosome strand and gene product. Gene product records contain the protein length, gene family attribute, PI, Mw, GI number and definition on NCBI, and mRNA coding and protein sequences. In addition, functional assignment, subcellular localization prediction and taxonomic distribution among different groups (archaea, bacteria, metazoans, fungi, plants, viruses and other eukaryotes) are provided for further study of the functional and evolutionary patterns of intronless genes. Some gene features are hyperlinked to their respective gene pages and to external sites and databases that provide more detailed information. In general, a cascading style is applied for data browsing, which is browsed in steps, starting with species, then providing an intronless gene list and finally single gene annotations (Figure 1).

### Comparison

**Figure 2:**
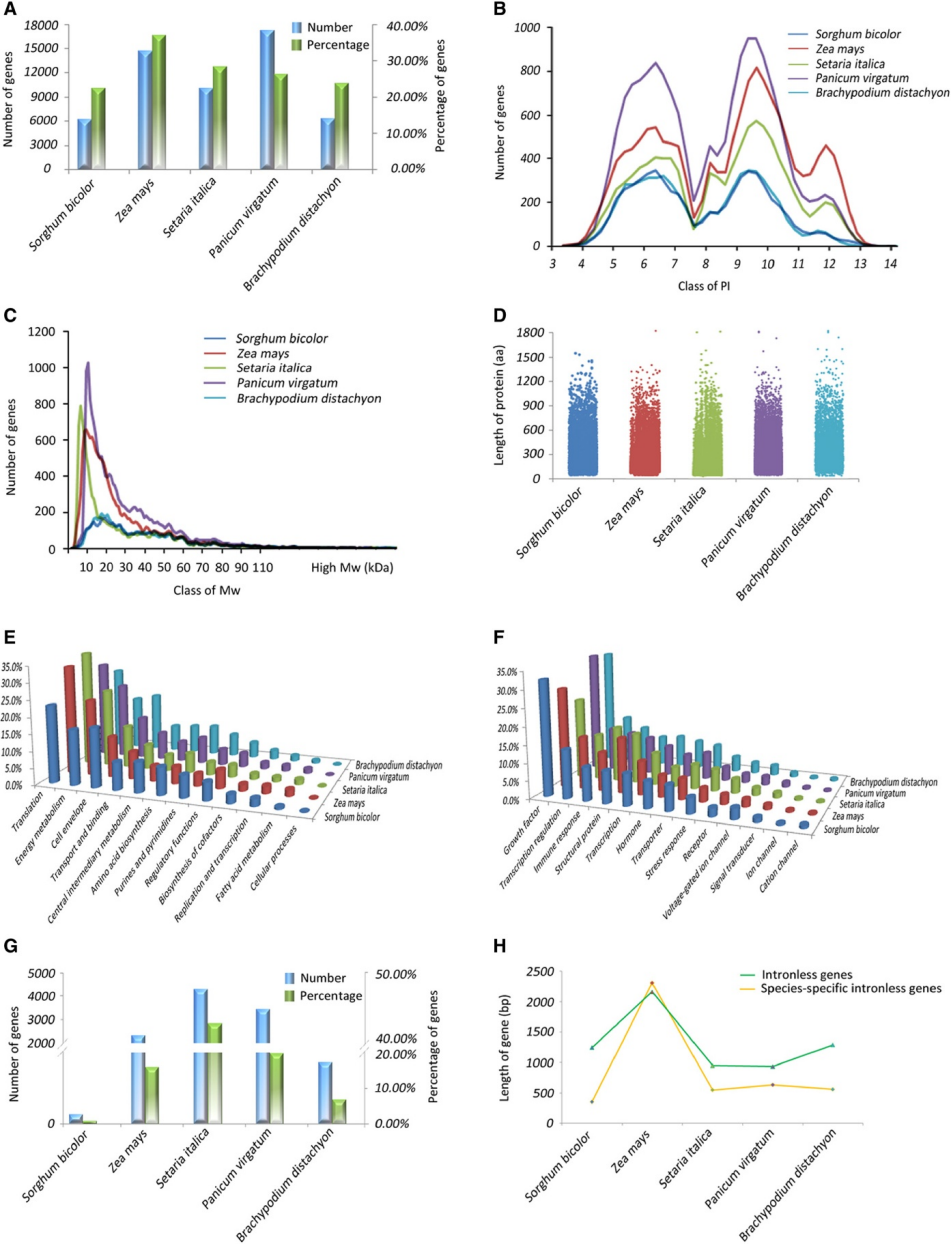
**Comparative analysis of intronless genes found in the five species included in the database: (A) the number and percentage of intronless genes; (B) isoelectric point; (C) molecular weight; (D) length of protein (number of amino acids); (E) cellular role; (F) Gene Ontology; (G) species-specific intronless genes on gene number and (H) their gene length.**

The results of comparative analysis among the five species are shown on Comparison interface. A comprehensive analysis of the genomes of *Sorghum bicolor*, *Zea mays*, *Setaria italica*, *Panicum virgatum* and *Brachypodium distachyon* showed that these consist of 22.45% (6,197), 36.87% (14,623), 28.23% (10,015), 26.15% (17,227) and 23.63% (6,274) intronless genes, respectively (Figure 2A). The variability in the number of intronless genes observed is clearly correlated with the size of the genome. A bimodal distribution of PI in proteins encoded by intronless genes was observed in all five Poaceae species, with a smaller third peak between two main peaks (Figure 2B). These results are consistent with those previously reported [25]. The distribution of the Mw of intronless genes was unimodal, with a peak value ranging from 7 to 13 kDa (Figure 2C). To investigate the general trends in protein length distribution, five scatter plots of protein length were generated (Figure 2D). The average protein length was approximately 337 amino acids (aa) in *Sorghum bicolor*, 222 aa in *Zea mays*, 215 aa in *Setaria italica*, 249 aa in *Panicum virgatum* and 331 aa in *Brachypodium distachyon*. To explore the biological function of proteins encoded by intronless genes, the cellular roles and GO categories of these genes were predicted and compared in each species. The results showed that the percentages of intronless genes that fell under the different functional categories were similar across species, with the largest number of intronless genes categorized as translation (T) and growth factor (GF) in the cellular role and GO categories, respectively (Figure 2E, F). In addition, the genic parameters for species-specific intronless genes were calculated, including gene number and length (Figure 2G, H). Overall, the comparative analyses of intronless genes among Poaceae species clearly showed several common functional and evolutionary characteristics that may also be found in other major eukaryote kingdoms. However, species-specific features remain and may represent one of the sources of the observed natural biodiversity.

### Download

A file transfer protocol (FTP) download server provides users the option to download files in bulk form.

### Help

To help address any specific problems or issues users may encounter, a detailed tutorial was developed. This tutorial introduces the user to the PIGD interface and is accessed from the “Help” drop-down menu. In addition, links to external databases have been provided. PIGD has a data submitting system via which we encourage researchers to submit data about intronless genes in other eukaryotic genomes. The database administrators will approve sequences and annotation information submitted by users before their inclusion into PIGD.

## Discussion

Recently, eukaryotic intronless genes have gained increasing attention because of their potential use in understanding evolutionary patterns of related genes and genomes. Relevant databases have been set up and have aided evolutionary and functional studies. For example, researchers identified six 10-nt motifs that are present in more than 90% of the intronless genes, using the IGD data. It is further hypothesized that one or more of these motifs function in intronless mRNA export and/or that a structure is involved [26]. However, to date, there is no such dedicated database for intronless genes in plants. Given the rapid advancement of next-generation sequencing, more and more plant genomes are being sequenced. Developing a plant intronless genes database could greatly aid research by data mining such valuable genomic resources. In PIGD, we offer a feature-rich and user-friendly integration of data, tools and analyses.

First, PIGD provides comparative genomic data for the Poaceae that permits the identification of features that are conserved or divergent during evolution (Figure 2). We used the characterization of species-specific intronless genes as an example. The study of species-specific genes has generated much interest in recent years because these genes are of particular importance for understanding plant adaptation. Comparative analysis of species-specific intronless genes showed the dramatic difference on their gene number among the five species. Meanwhile, Yang et al. [27] compared specific genes identified in plants and animals, and found that there are many more specific genes in plant genomes. This difference may suggest that gene duplication followed by rapid evolution occurred in plants at a higher frequency [28]. Detailed information on species-specific intronless genes makes it possible for the user to investigate codon usages, syntenic genomic regions and molecular comparison, thereby help the user to determine the exact mechanisms regarding the origin of species-specific genes.

Second, popular tools such as NCBI BLAST are embedded in our website to help annotate new sequences and predict putative orthologous relationships of genes. Also, PIGD provides an interactive platform for communication and data sharing among members of the same research team, where researchers can download the available data and submit their important scientific findings.

The PIGD database will be updated at least once a year, and the number of available plant species included will be expanded. Future versions of the database are expected to include detailed gene annotations, such as RNA-seq data, paralogs and KEGG ontology, to enable researchers to further explore the function and evolution of intronless genes. Meanwhile, powerful comparative analysis tools of intronless genes in plants will be developed to provide a centralized platform that could benefit a wide variety of related research.

## Conclusion

PIGD is the only existing plant intronless genes database that complements the IGD database for human intronless genes. The availability of online sequence databases provides an easy entry point for researchers to immediately access information about intronless genes, without the need to install additional software. Furthermore, statistical analyses relevant to genetic features and functional classification systems are provided in both databases. Such integration and analysis of data may help users understand the biological role of intronless genes in the genomes of higher organisms and explore the functionality of introns in gene evolution.

## Availability and requirements

PIGD is freely accessible at http://pigd.ahau.edu.cn/. Moreover, all data are available for download in flat file form from the FTP sites. Inquiries concerning the database may be directed to PIGD_MKL@163.com. Operating system(s): Platform independent.Programming languages: PHP, MySQL, HTML and JavaScript. License: Not required. Any restrictions to use by non-academics: None.

## Abbreviations

PIGD: Poaceae Intronless Genes Database
GPCRs: G-protein–coupled receptors
SAURs: Small auxin–up RNAs
PI: Isoelectric point
Mw: Molecular weight
GO: Gene Ontology
T: Ttranslation
GF: Growth factor.
